# Supplementary material for: In-vivo integration of soft neural probes through high-resolution printing of liquid electronics on the cranium
Source: Nat Commun. 2024 Feb 27;15:1772. doi: 10.1038/s41467-024-45768-0 (PMC10899244; doi:10.1038/s41467-024-45768-0)
Supplement: Supplementary file 1 — Supplementary Information [file 41467_2024_45768_MOESM1_ESM.pdf]

In-vivo integration of soft neural probes through high-resolution printing of liquid electronics on the cranium

Young-Geun Park[1,2†], Yong Won Kwon[1,2†], Chin Su Koh[3†], Enji Kim[1,2†], Dong Ha Lee[1,2], Sumin Kim[1,2], Jongmin Mun[4], Yeon-Mi Hong[1,2], Sanghoon Lee[1,2], Ju-Young Kim[2,5], Jae-Hyun Lee[2,5], Hyun Ho Jung[3★], Jinwoo Cheon[2,5,6★], Jin Woo Chang[7★], Jang-Ung Park[1,2,3,5★].

[1]Department of Materials Science and Engineering, Yonsei University, Seoul 03722, Republic of Korea

[2]Center for Nanomedicine, Institute for Basic Science (IBS), Seoul 03722, Republic of Korea

[3]Department of Neurosurgery, Yonsei University College of Medicine, Seoul 03722, Republic of Korea

[4]Department of Statistics and Data Science, Yonsei University, Seoul 03722, Republic of Korea

[5]Graduate Program of Nano Biomedical Engineering (NanoBME), Advanced Science Institute, Yonsei University, Seoul 03722, South Korea.

[6]Department of Chemistry, Yonsei University, Seoul 03722, South Korea.

[7]Department of Neurosurgery, Korea University Anam Hospital, Seoul 02841, South Korea.

★e-mail: jang-ung@yonsei.ac.kr (J.-U. P.); jchang@yuhs.ac (J. W. C.); jcheon@yonsei.ac.kr (J. C.); junghh@yuhs.ac (H. H. J.)

† These authors contributed equally to this work.

This Supplementary Information includes:

Supplementary Figures

1. Sweeping test of Loctite.
2. Cytotoxicity test of Loctite 4011 using both Loctite 4011 samples (i) with the printed EGaIn lines and (ii) without EGaIn lines.
3. Block diagram of a cranial circuit for testing of wireless data transfer across the scalp skin.
4. Schematic illustration and photographs showing the operation of wireless data transfer from cranial circuit to a smartphone.
5. Representative LFP traces recorded by the NFC-based system.
6. A schematic illustration and a 3D-reconstructed image acquired by micro-CT scan of the mouse calvaria 6 weeks after the circuit formation.
7. Neural probes, cranial interconnection, and a circuit diagram.
8. Mechanical compression test of printed EGaIn circuit.
9. Accelerated aging test of EGaIn.
10. Signal quality test with in-vitro setup mimicking the signal recording process from the brain to analyzer.
11. Representative recorded single-unit traces and signal-to-noise ratios from conventional Nichrome probes with PCB connectors and our soft neural probe with EGaIn interconnections.
12. 4 different structures of conformal neural recording circuits with stereotaxic coordinates.
13. Representative single-unit traces recorded by the wi-fi-based system.
14. Implantation of neural probes.

15. Optical stereomicrographs showing the implanted neural probes and their outer end exposed on the cranial surface.
16. Optical micrograph of a soft neural probe implanted in the brain tissue of mouse. For the imaging, the brain was cleared using SHIELD protocols
17. Schematic illustrations showing the process of monolithic integration of implanted neural probes.
18. Single-unit recording yield and single-unit spike clusters among 12 soft neural probes over 33 weeks.
19. Time-evolution plots of PCA-clustered single-unit spikes in channels 2, 5, 7, and 9 over 33 weeks after injection.
20. Time evolution of single-unit spikes clustered from channel 10 over 33 weeks after injection.
21. Color-blind safe images of 3D-reconstructed confocal micrograph and fluorescence micrograph for Figure 4i and m.
22. MTT and Calcein assays of SH-SY5Y cells cultured in media pre-contained with the pristine EGaIn and PtB/EGaIn samples.
23. Fluorescence micrographs of a horizontal section of the mouse brain 8 weeks after implantation with our soft neural probe by capillary-assisted injection.
24. Histology images of the hematoxylin and eosin (H&E) stained scalp skin of a mouse 6 weeks after the cranial circuit formation.
25. Schematic illustration of the shape and size of T-maze.
26. Representative single-unit traces and spike raster of the hippocampus CA1 and the primary visual cortex L6 regions during the T-maze test.
27. Hippocampal signals recorded from another mouse.

28. Motion-related signals from visual cortex of another mouse.

Supplementary Table

1. Comparison of our neural interface system to state-of-art neural probes.

Supplementary Figures

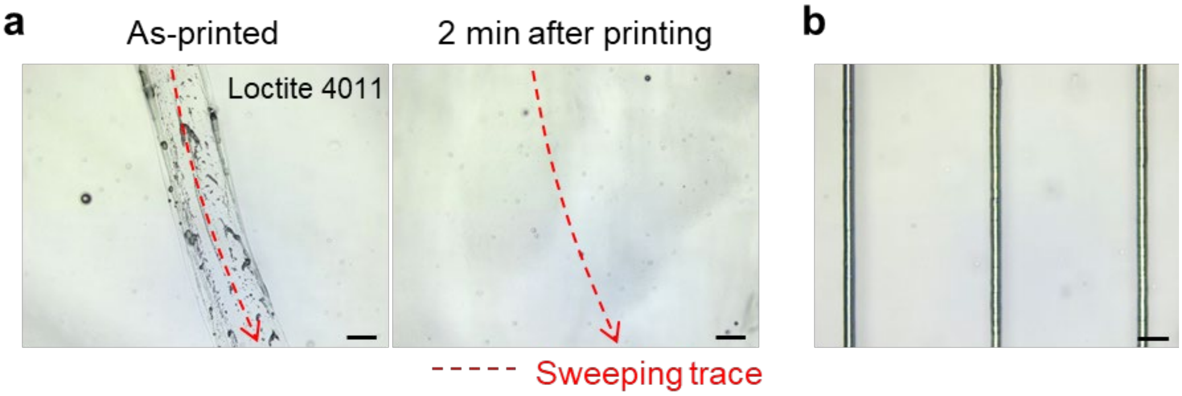

Supplementary Fig. 1. Sweeping test of Loctite. **a,** Optical micrographs of the 30 µm-thick Loctite 4011 samples for a sweeping test. Scale bars, 200 µm. **b,** Optical micrograph of EGaIn lines printed directly on this cured Loctite layer (thickness: 30 µm, curing time: 2 minutes). Scale bar, 100 µm. In order to verify the mechanical curing of this thin Loctite layer (thickness: 30 µm), a sweeping test was conducted after printing the 30 µm-thick layer of Loctite 4011 on a glass substrate. This Loctite layer was then swabbed at different time points from the as-printed state to 2 minutes after printing. Sweeping traces of chafing were observed immediately after Loctite printing until 30 seconds after printing, indicating that curing was not complete. However, after more than 1 minute, there was no damage to the Loctite 4011 layer by swabbing, indicating that it was cured well enough to allow further processes to form the cranial electronics, including EGaIn printing, top encapsulation, and suturing. The experiment was repeated 3 times with similar results.

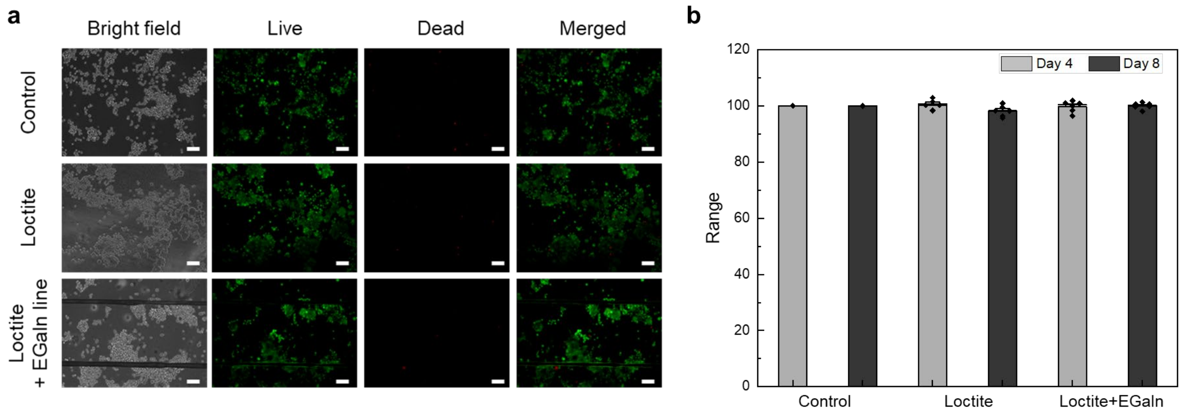

Supplementary Fig. 2. Cytotoxicity test of Loctite 4011 using both Loctite 4011 samples (i) with the printed EGaIn lines and (ii) without EGaIn lines. **a,** Live/dead assay of Neuro2a cells cultured (i) on a glass slide (control), (ii) on a Loctite 4011 layer, and (iii) on Loctite 4011 with the printed EGaIn lines, after 8 days of incubation, respectively. Scale bars, 50 µm. **b,** Quantitative analysis of the Live/Dead assay after 4, 8 days of cultivation (n=6). Error bars represent standard error.

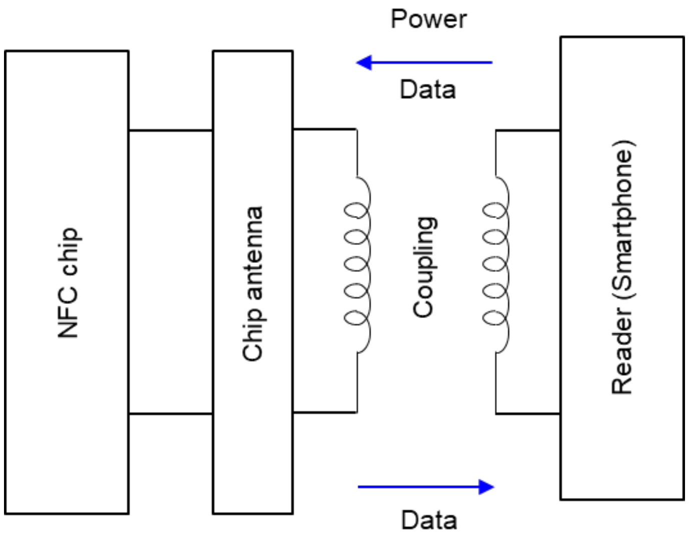

Supplementary Fig. 3. Block diagram of a cranial circuit for testing of wireless data transfer across the scalp skin.

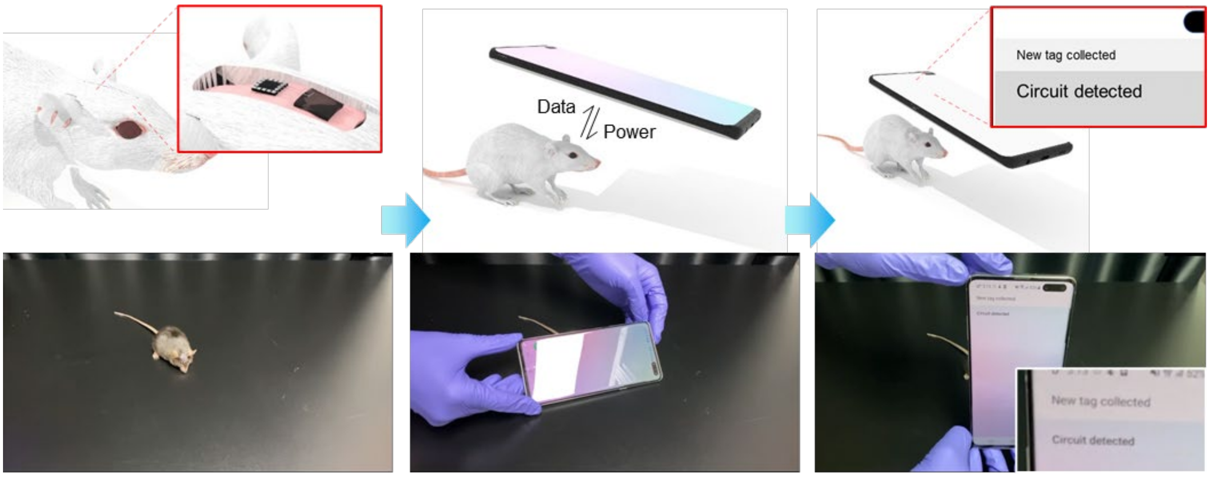

Supplementary Fig. 4. Schematic illustration and photographs showing the operation of wireless data transfer from cranial circuit to a smartphone. The experiment was repeated 3 times with similar results.

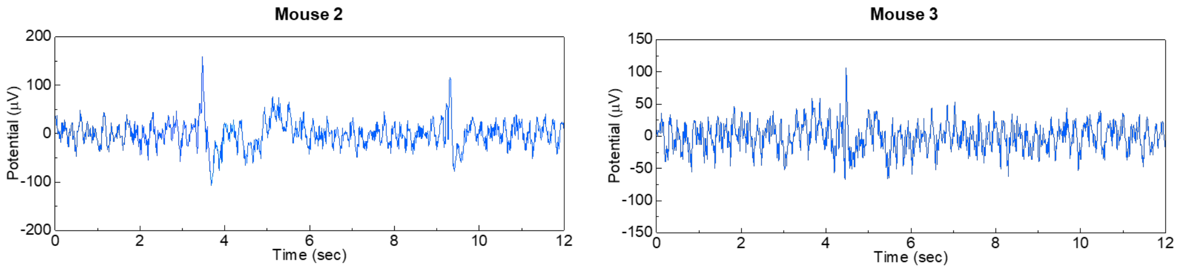

Supplementary Fig. 5. Representative LFP traces recorded by the NFC-based system using two different mice (out of three). The result of the other mouse (mouse 1) was presented in Fig. 2h.

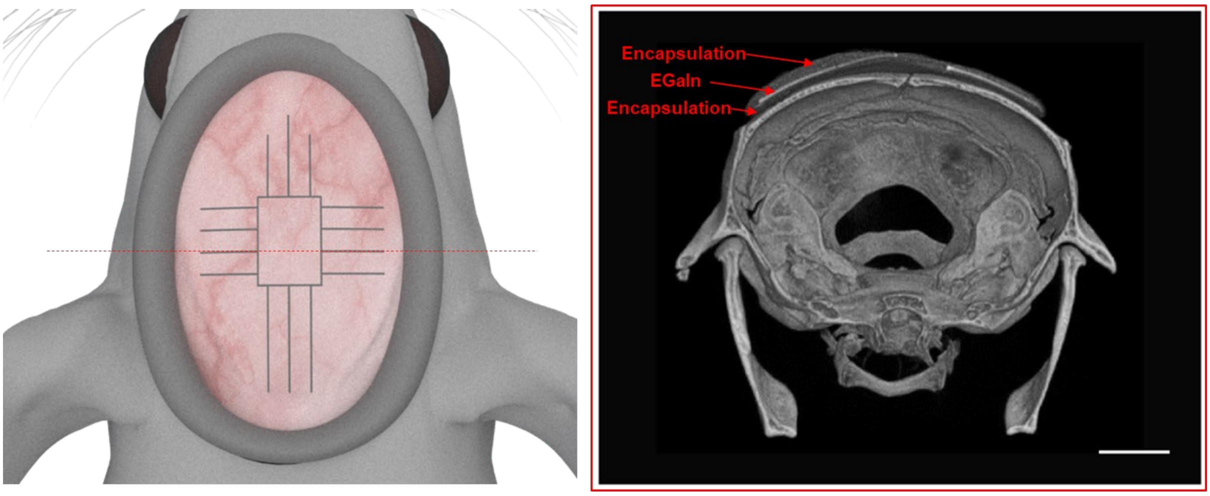

Supplementary Fig. 6. A schematic illustration showing the top-view of our printed circuit pattern for micro-CT (left), the 3D-reconstructed image acquired by micro-CT scan of the mouse calvaria 6 weeks after this circuit formation. Scale bar, 2 mm (right).

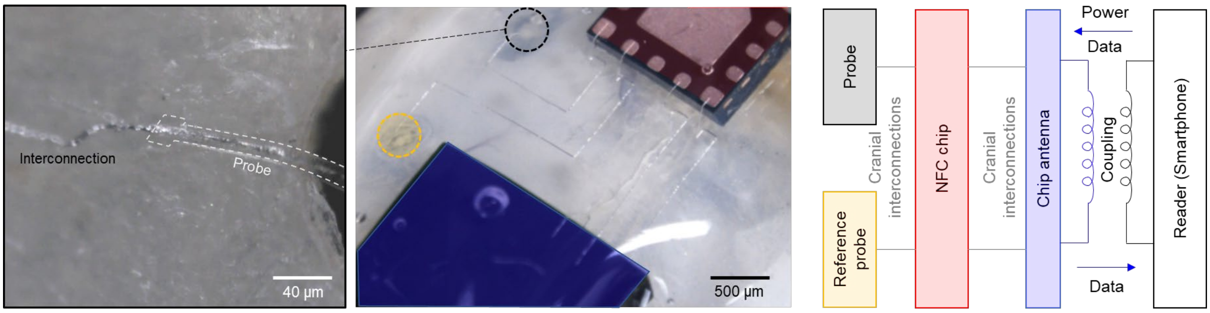

Supplementary Fig. 7. A photograph of the conformal wireless neural recording system with soft neural probes, a wireless recording unit, a chip antenna, and their interconnections on the cranium (middle), a magnified photograph showing the connection between the neural probe and cranial interconnection (left), a diagram on this neural recording system with corresponding colors of components in the photograph (right).

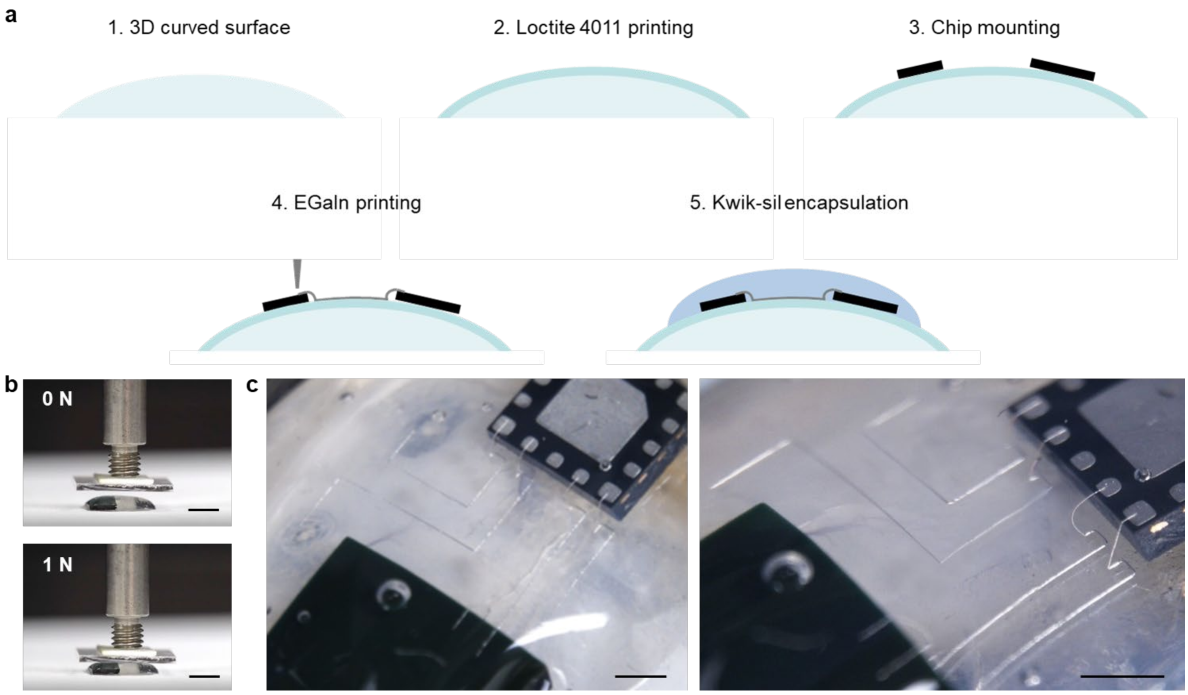

Supplementary Fig. 8. Mechanical compression test of printed EGaIn circuit. **a,** Schematic illustration showing the entire fabrication procedure of a sample for mechanical compression test. **b,** Photographs of the printed wireless circuitry captured before and during compression at 1 N. Scale bars, 5 mm. **c,** Stereomicrographs before (left) and after (right) this mechanical compression. Scale bars, 1 mm.

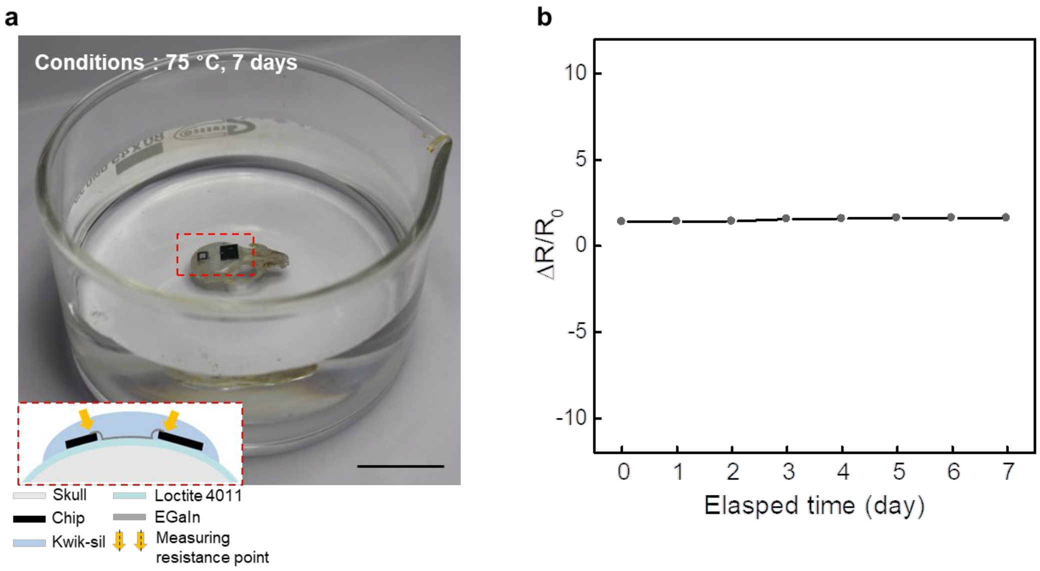

Supplementary Fig. 9. Accelerated aging test of EGaIn. **a,** Photograph of an NFC-based wireless circuit on the cranium immersed in a PBS solution for the accelerated aging condition. Inset: a schematic illustrating side view of this circuit. Scale bar, 2 cm. **b,** Relative change in electrical resistance of this sample for 7 days.

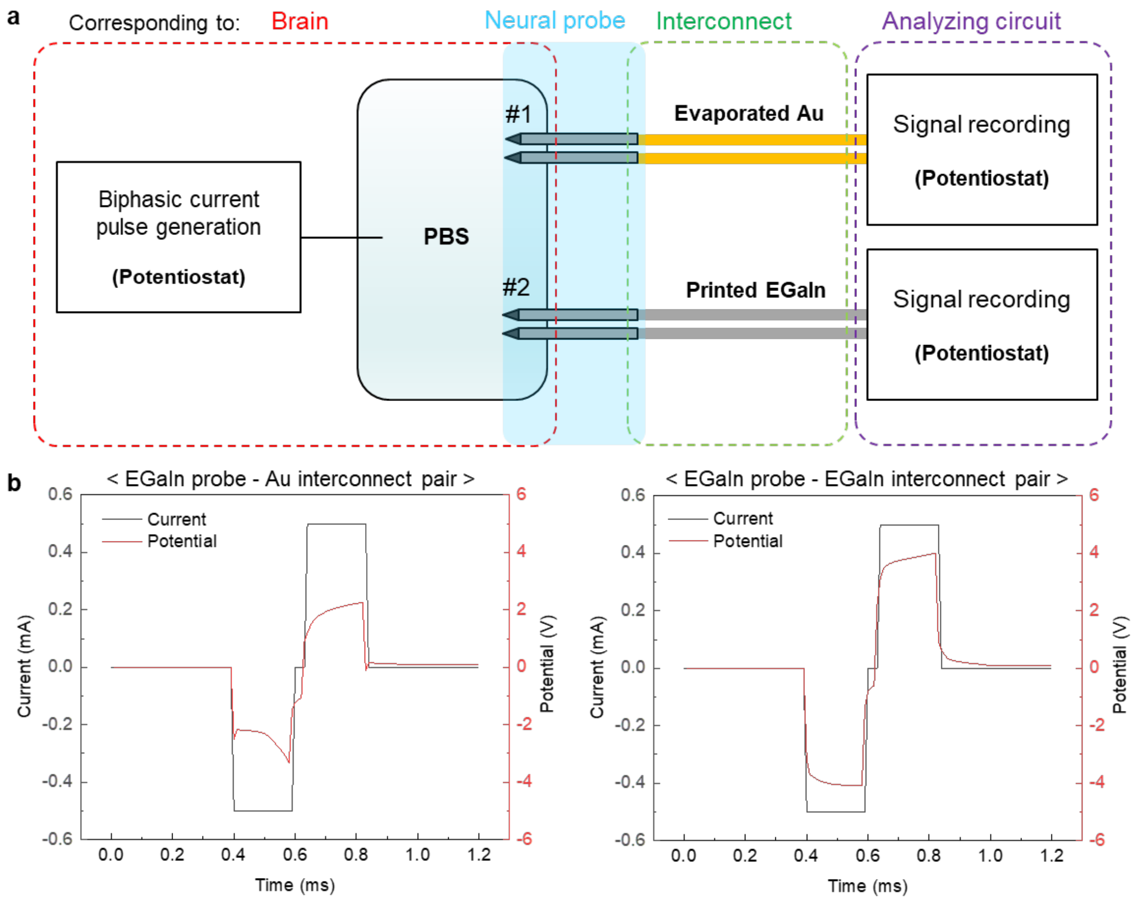

Supplementary Fig. 10. Signal quality test with in-vitro setup mimicking the signal recording process from the brain to analyzer. **a,** Schematic illustration of the experimental setup for the signal quality test. **b,** Plots of acquired potential waveforms through a heterogeneous (soft neural probe – evaporated Au) pair (left) and through a monolithic (soft neural probe – printed EGaIn) pair (right).

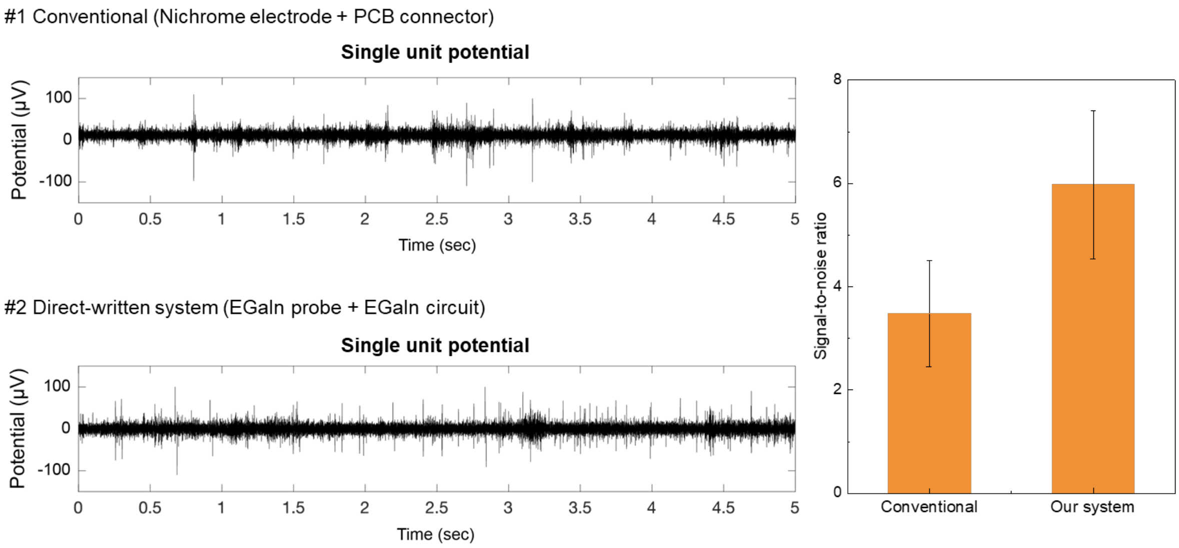

Supplementary Fig. 11. Representative recorded single-unit traces and signal-to-noise ratios from (i) conventional Nichrome probes with PCB connectors (top) and (ii) our soft neural probe with EGaIn interconnections (bottom). Both systems were each interfaced with a wi-fi module for data transmission. Signals were recorded from mice 4 weeks after implantation. Each error bar in signal-to-noise ratio plot represents a standard deviation of measurements from three different mice.

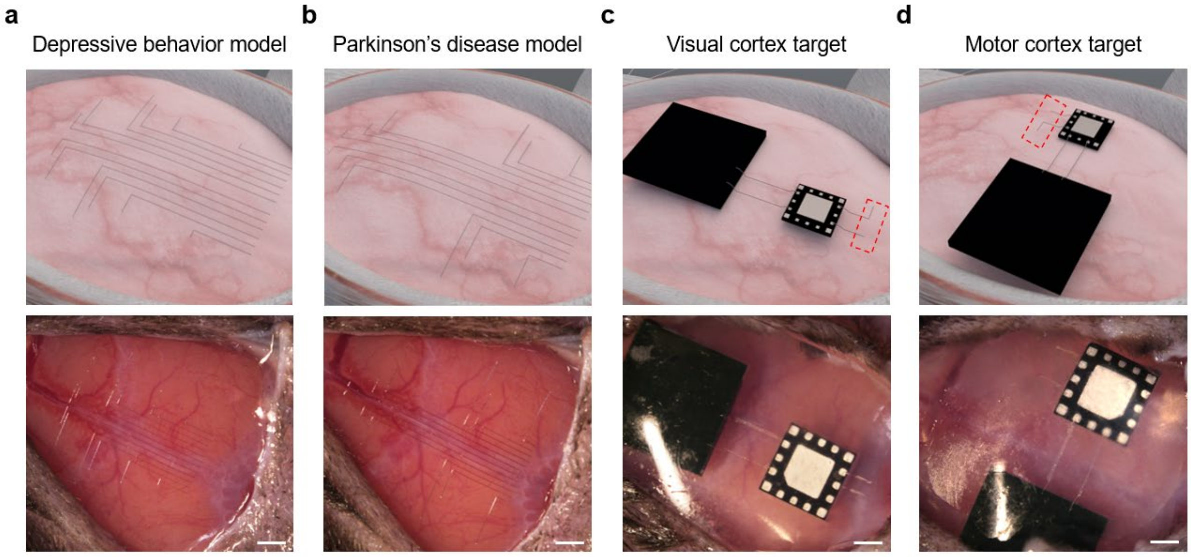

Supplementary Fig. 12. Schematic illustrations (top) and stereomicrographs (bottom) describing 4 different structures of conformal neural recording circuits targeting **a,** depressive behavior model with stereotaxic coordinates: (1) Medial prefrontal cortex (mPFC): 2.5 mm AP, ±0.25 mm ML, -2.5 mm DV; 1.7 mm AP, ±0.75 mm ML, -2.5 mm DV. (2) Nucleus accumbens (NAc): 1.3 mm AP, ±1.6 mm ML, -4.4 mm DV. (3) Amygdala (AMY): -1.6 mm AP, ±3.1 mm ML, -4.9 mm DV. (4) Hippocampus (HIP): -2.0 mm AP, ±2.0 mm ML, -1.5 mm DV; -3.7 mm AP, ±3.0 mm ML, -4.8 mm DV, **b,** Parkinson's disease model with stereotaxic coordinates: (1) Motor cortex (MO): 0.8 mm AP, ±0.75 mm ML, -1.0 mm DV; 0.7 mm AP, ±1.8 mm ML, -1.6 mm DV. (2) Striatum: -0.4 mm AP, ±2.5 mm ML, -3.2 mm DV. (3) Globus pallidus internus (GPi): -0.7 mm AP, ±1.8 mm ML, -3.95 mm DV. (4) Subthalamic nucleus (STN): -1.4 mm AP, ±1.75 mm ML, -4.5 mm DV, **c,** visual cortex with a NFC-based wireless recording unit [stereotaxic coordinates: -3.1 mm AP, -0.9 mm ML, -0.6 mm DV; -3.1 mm AP, 1.1 mm ML, -0.6 mm DV], and **d,** motor cortex with a NFC-based wireless recording unit [stereotaxic coordinates: 0.7 mm AP, 1.8 mm ML, -1.6 mm DV; 0.8 mm AP, -0.1 mm ML, -1.0 mm DV]. Scale bars, 1 mm.

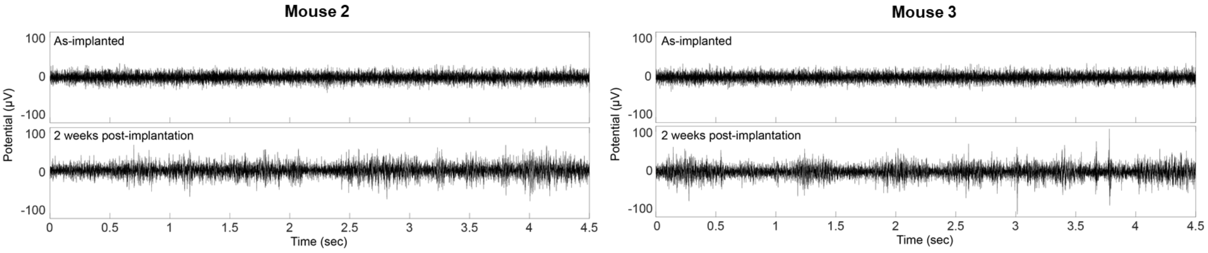

Supplementary Fig. 13. Representative single-unit traces recorded by the wi-fi-based system using two different mice (out of three). The result of the other mouse (mouse 1) was presented in Fig. 2j.

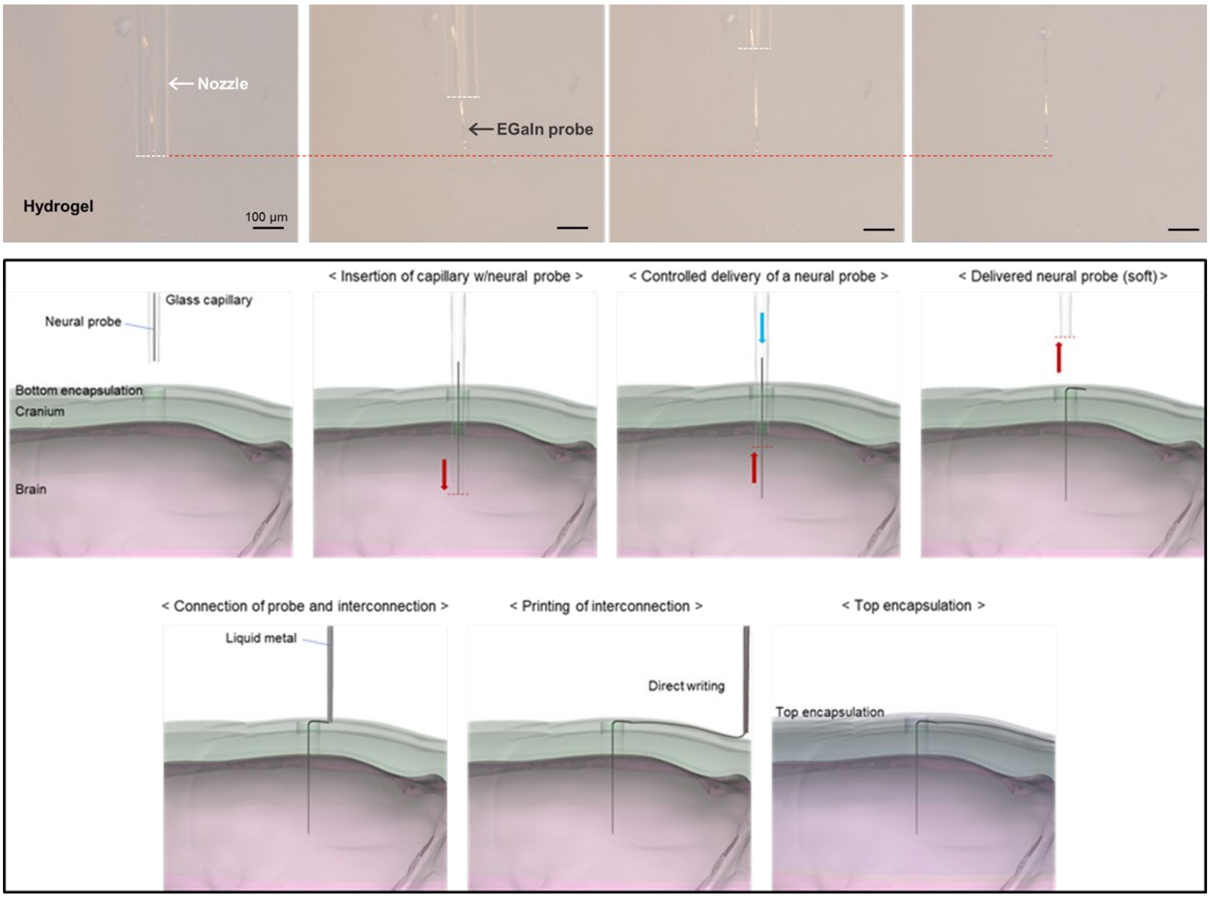

Supplementary Fig. 14. Sequential snapshots of our neural probes released from a glass capillary with matching the retraction velocity and volumetric flow rate (top). This capillary moved upward, while the end of the probe remained stationary (red dashed line). As a result, the neural probe maintained a consistent position with a displacement of less than 5 µm. In this experiment, 0.6% agarose gel was used as a brain phantom. The experiment was repeated 5 times with similar results. Scale bars, 100 µm. Schematic illustrations describing the procedures to implant a soft neural probe with a capillary and to connect the probe using liquid-metal interconnections (bottom).

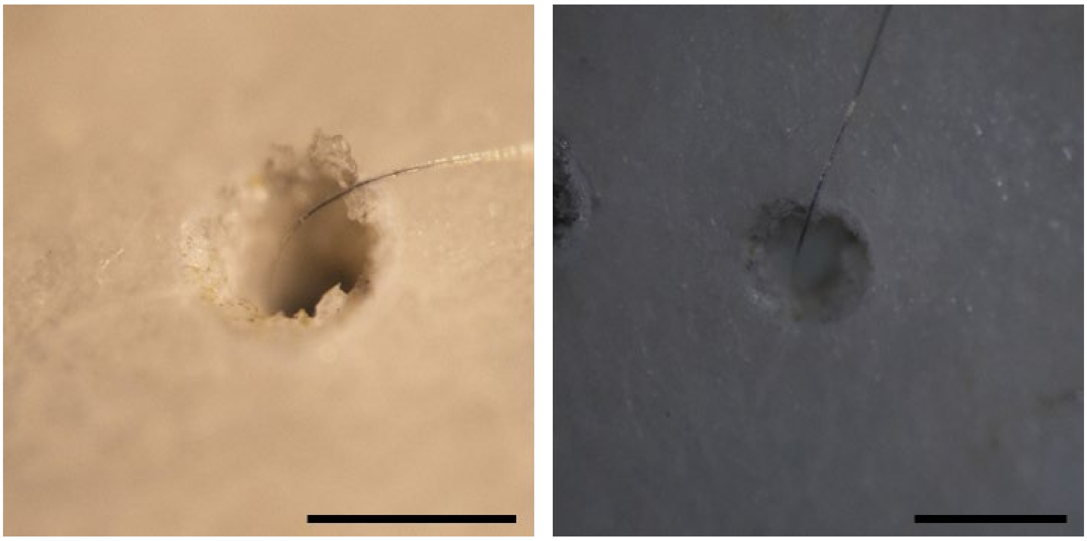

Supplementary Fig. 15. Optical stereomicrographs showing the implanted neural probes and their outer end exposed on the cranial surface. The experiment was repeated 30 or more times with similar results, including the implantation of neural probes for multi-channel recordings. Scale bars, 500 μm.

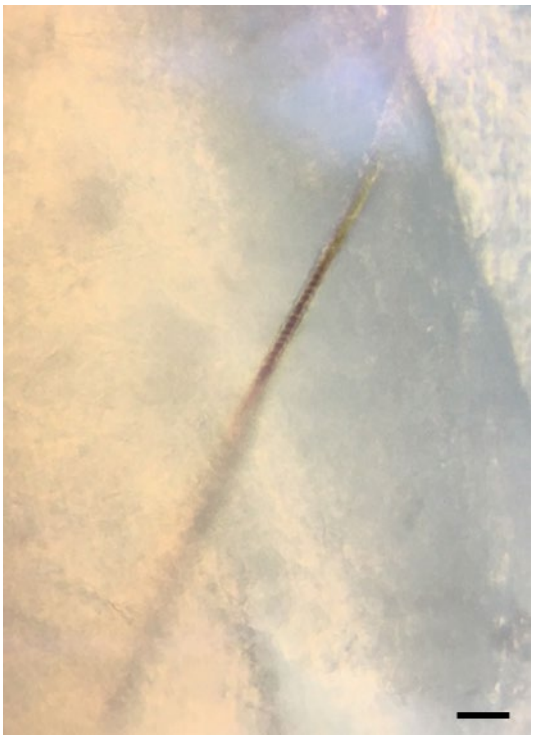

Supplementary Fig. 16. Optical micrograph of a soft neural probe implanted in the brain tissue of mouse. For the imaging, the brain was cleared using SHIELD protocols. Scale bar, 20 µm.

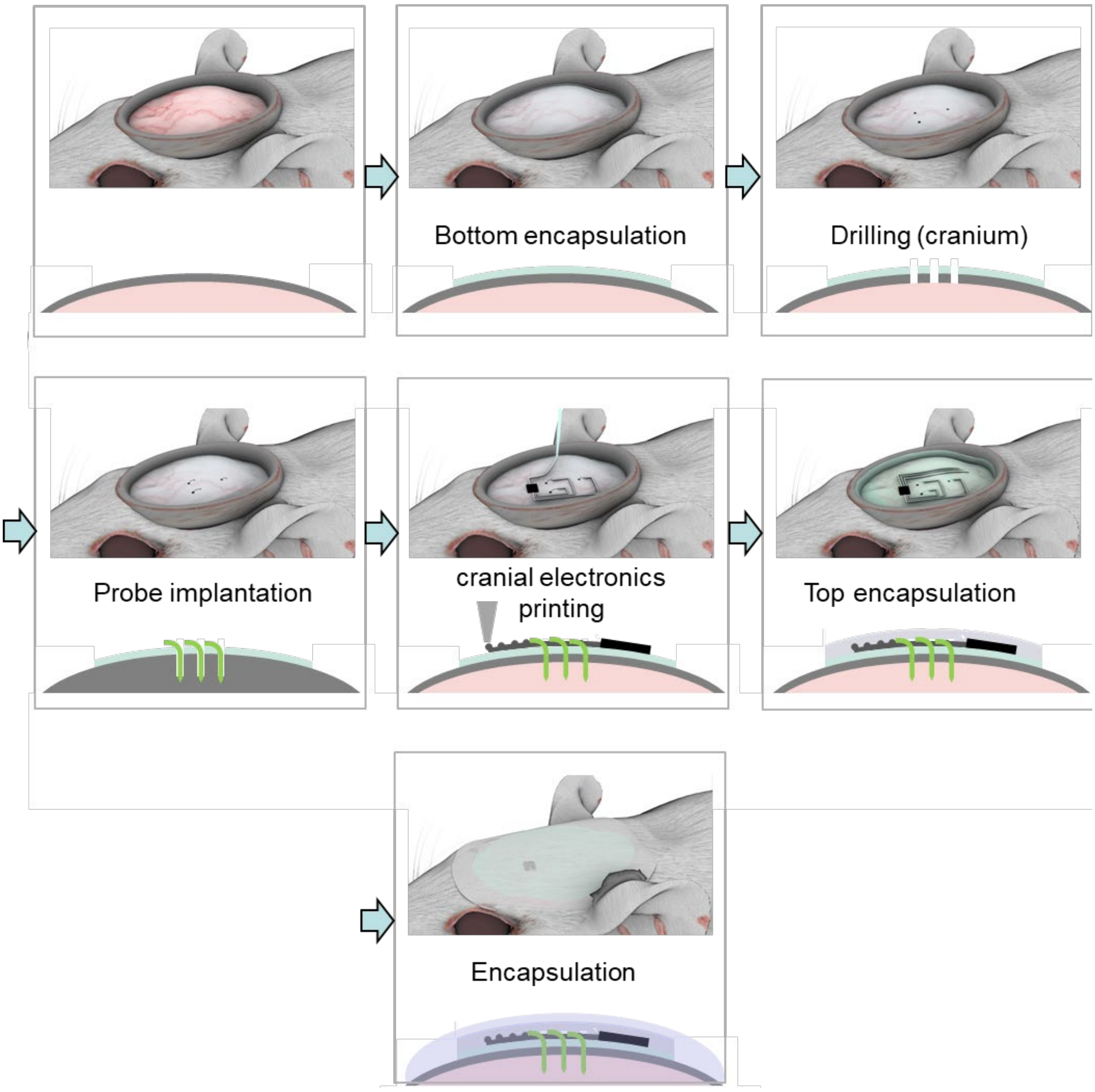

Supplementary Fig. 17. Schematic illustrations showing the process of monolithic integration of implanted neural probes.

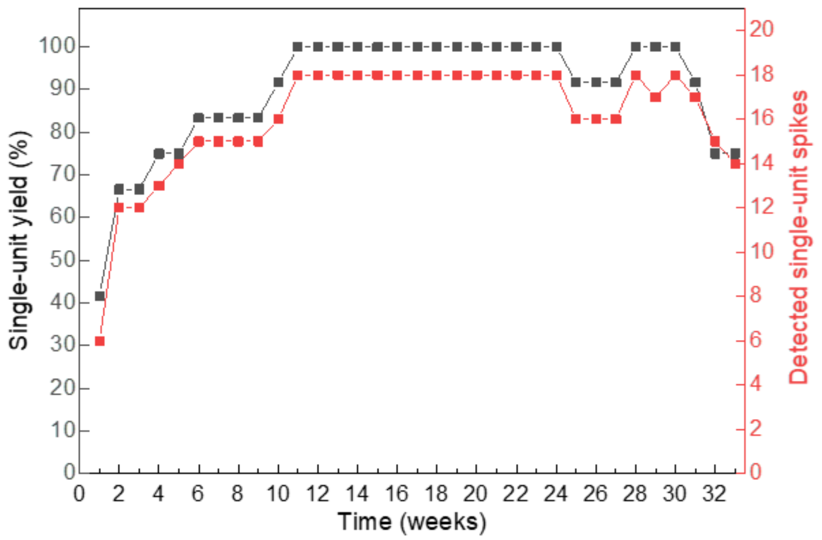

Supplementary Fig. 18. Single-unit recording yield and single-unit spike clusters among 12 soft neural probes over 33 weeks.

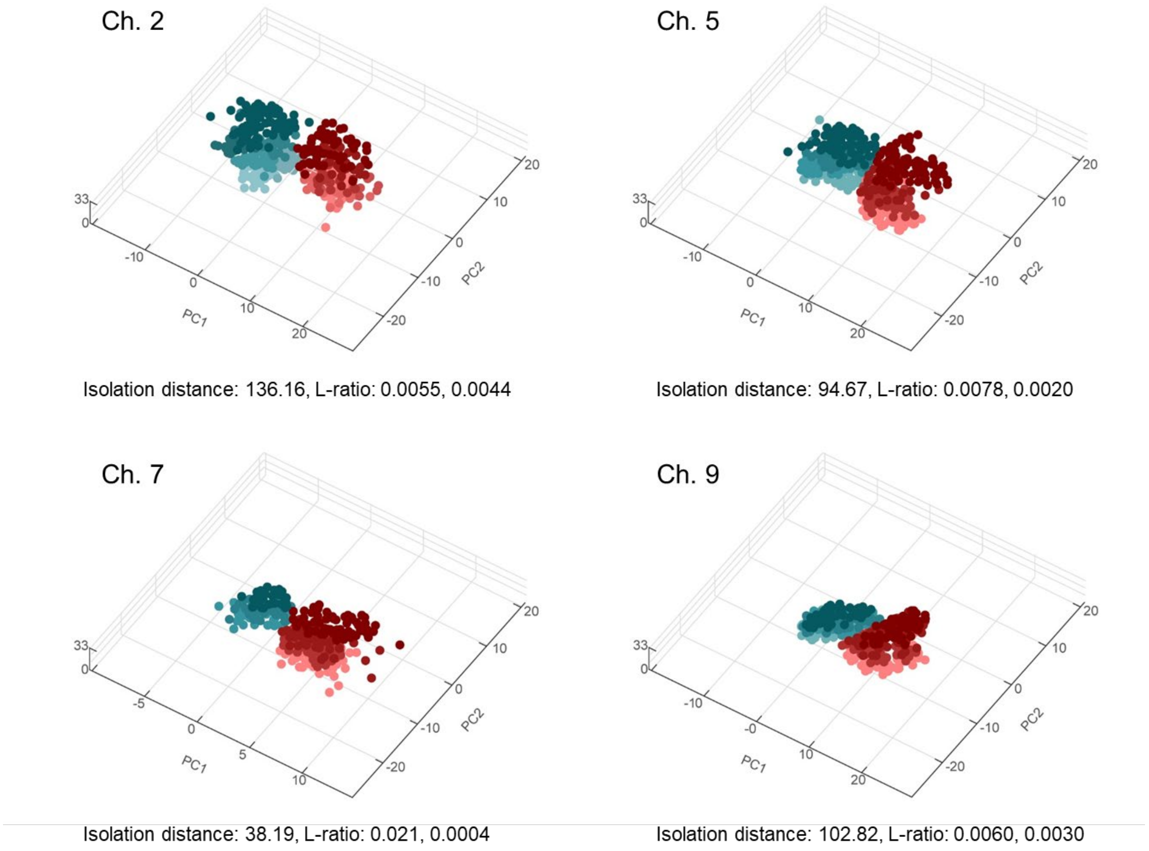

Supplementary Fig. 19. Time-evolution plots of PCA-clustered single-unit spikes in channels 2, 5, 7, and 9 over 33 weeks after injection. Isolation distance and L-ratios were measured at 16 weeks post-implantation.

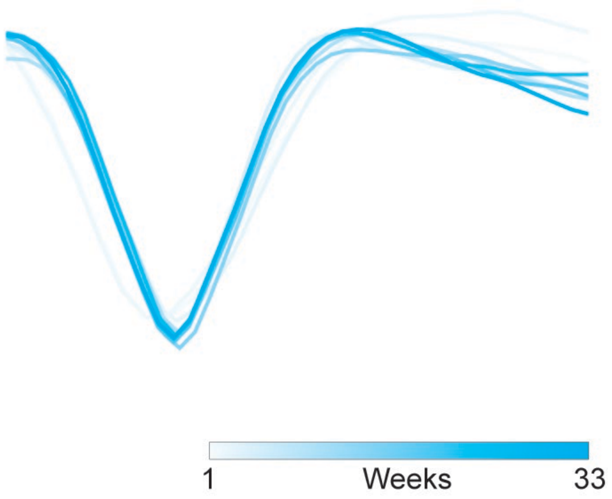

Supplementary Fig. 20. Time evolution of single-unit spikes clustered from channel 10 over 33 weeks after injection. Each spike is normalized to the maximum potential to show the similarity between waveforms.

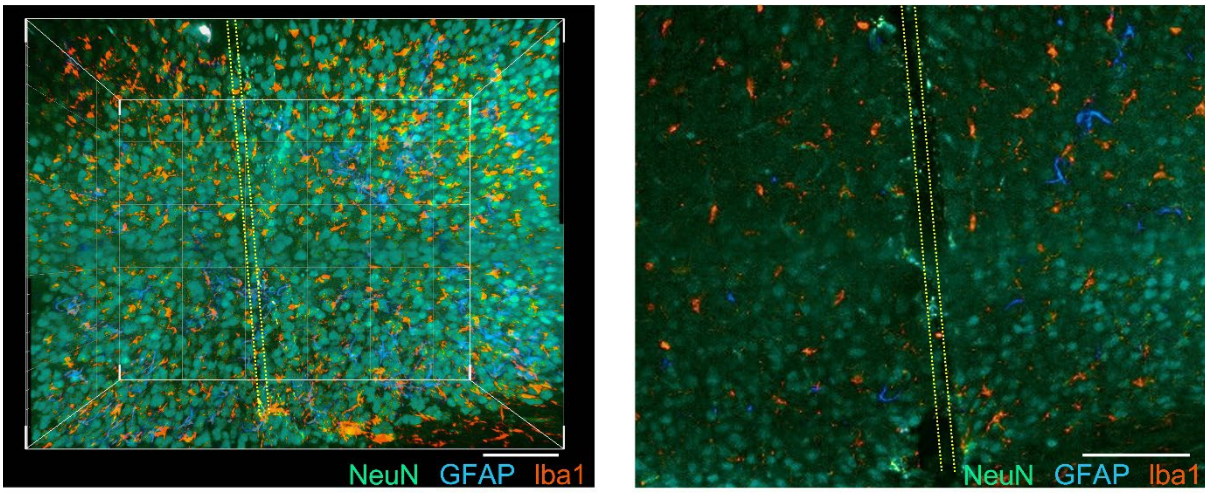

Supplementary Fig. 21. Color-blind safe images of 3D-reconstructed confocal micrograph and fluorescence micrograph showing the probe implanted in the hippocampal region. Scale bars, 100 μm.

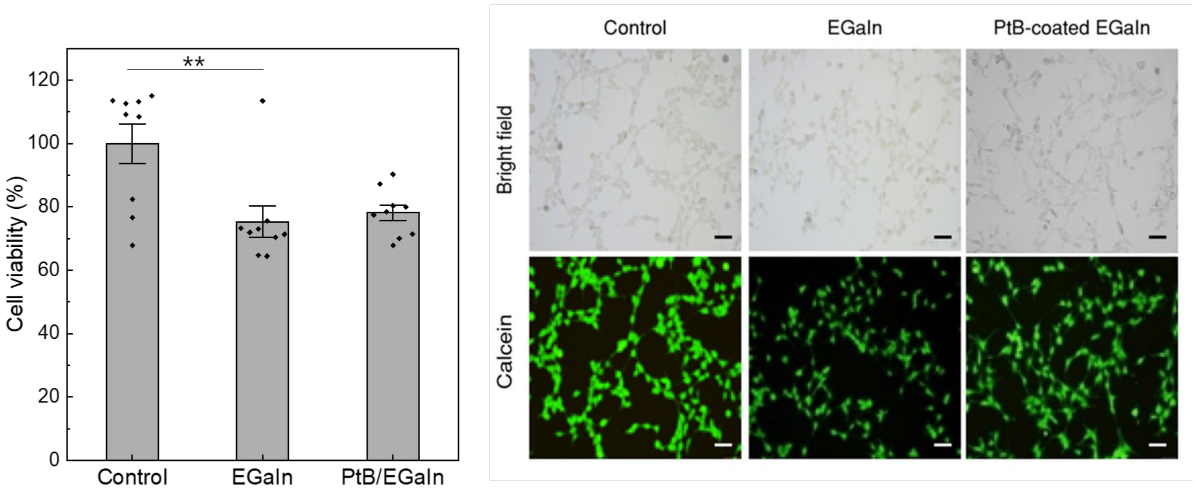

Supplementary Fig. 22. MTT and Calcein assays of SH-SY5Y cells cultured in media pre-contained with the pristine EGaIn and PtB/EGaIn samples for 7 days. Scale bars, 50 µm. Error bars represent standard error (paired two-tailed t-test: $^{**}p = 0.007$) (n=9).

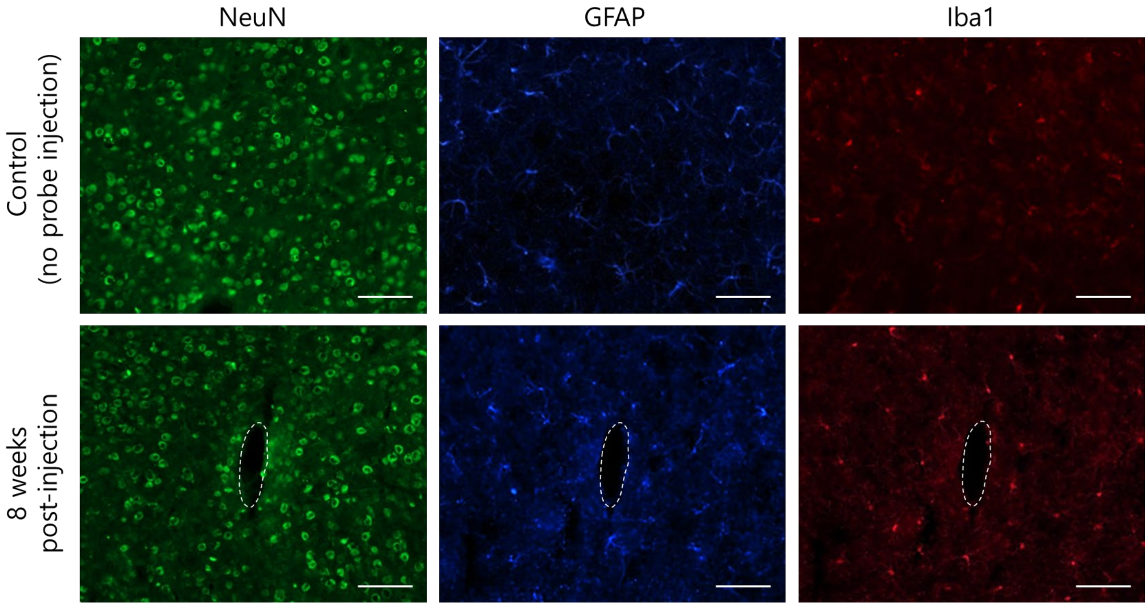

Supplementary Fig. 23. Fluorescence micrographs of a horizontal section of the mouse brain 8 weeks after implantation with our soft neural probe by capillary-assisted injection, compared to the control sample with no probe injection. Neurons, astrocytes, and microglia were stained with NeuN (green), GFAP (blue), and Iba1 (red), respectively. Scale bars, 50 μm. The experiment was repeated 6 times with similar results.

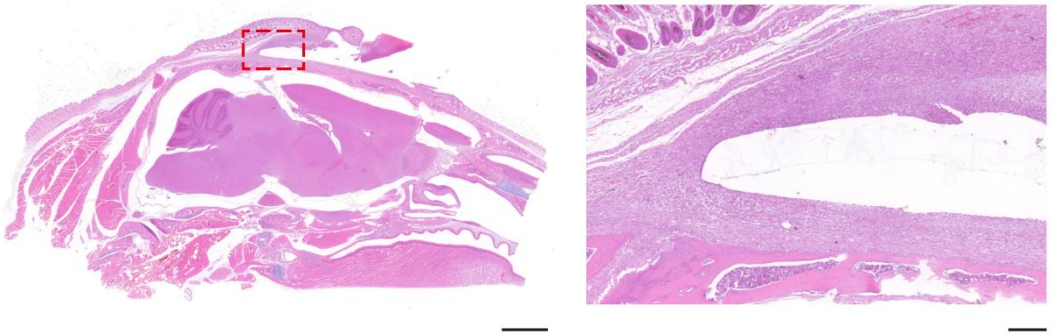

Supplementary Fig. 24. Histology images of the hematoxylin and eosin (H&E) stained scalp skin of a mouse 6 weeks after the cranial circuit formation. Scale bars, 2 mm (left), 200 µm (right).

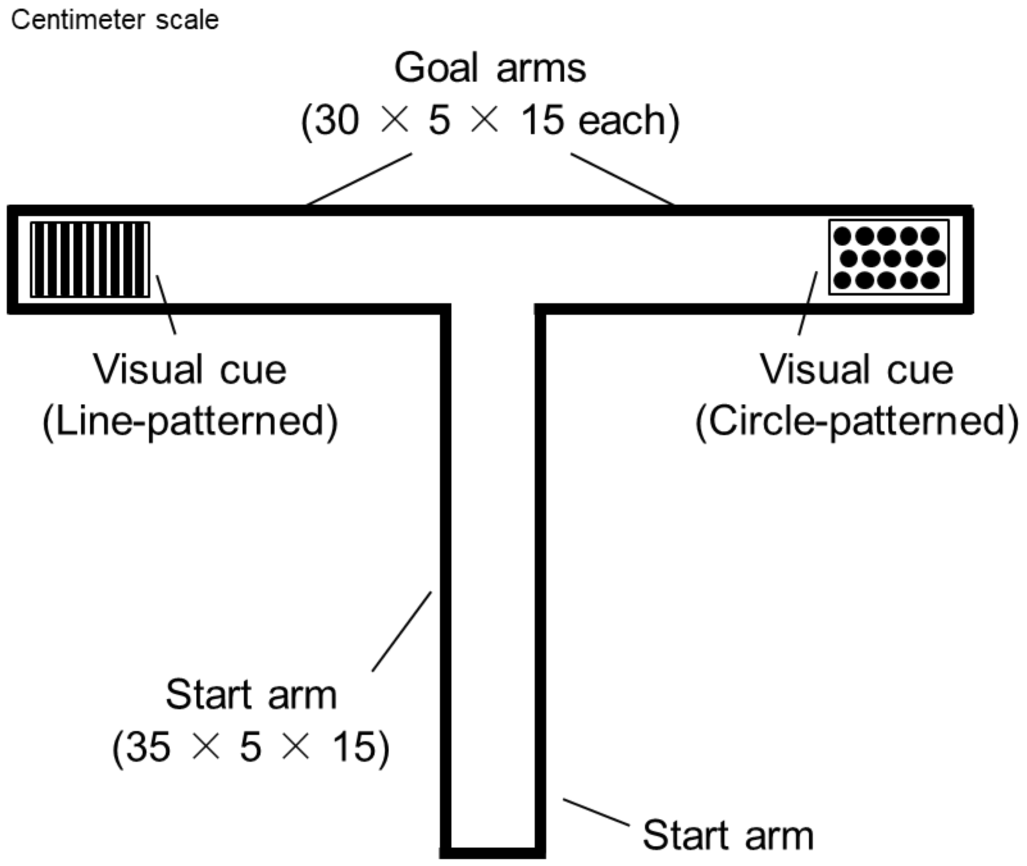

Supplementary Figure 25. Schematic illustration showing the shape and size of T-maze.

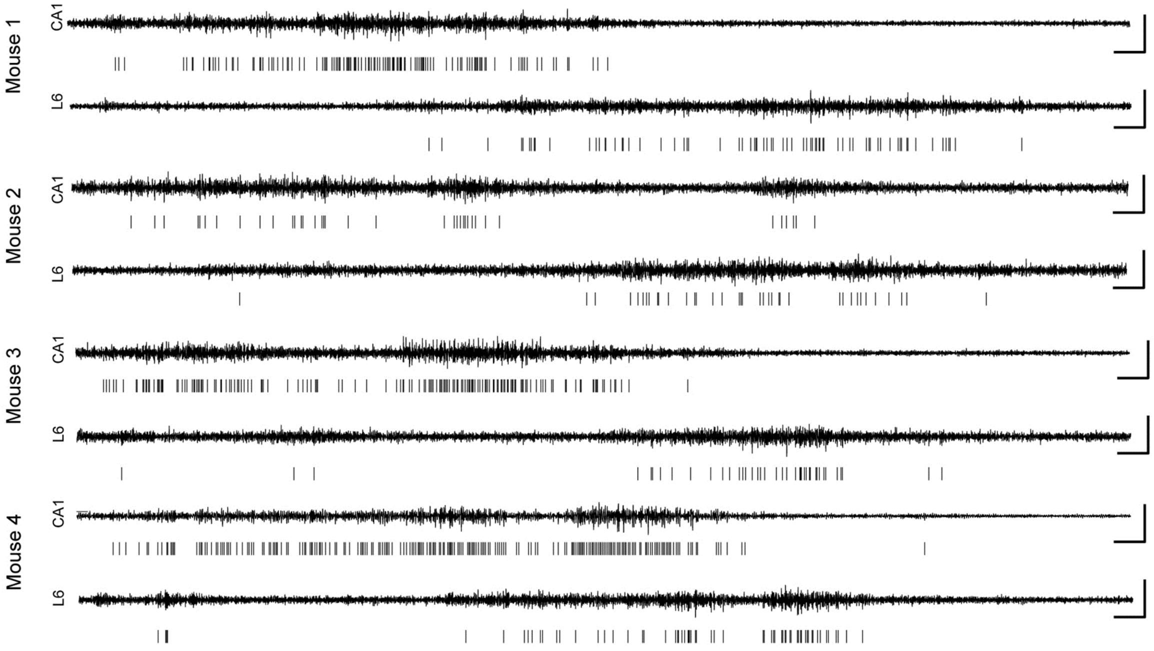

Supplementary Fig. 26. Representative single-unit traces and spike raster of the hippocampus CA1 and the primary visual cortex L6 regions during the T-maze test using different mice. Scale bars, 300 μV (vertical) and 100 ms (horizontal).

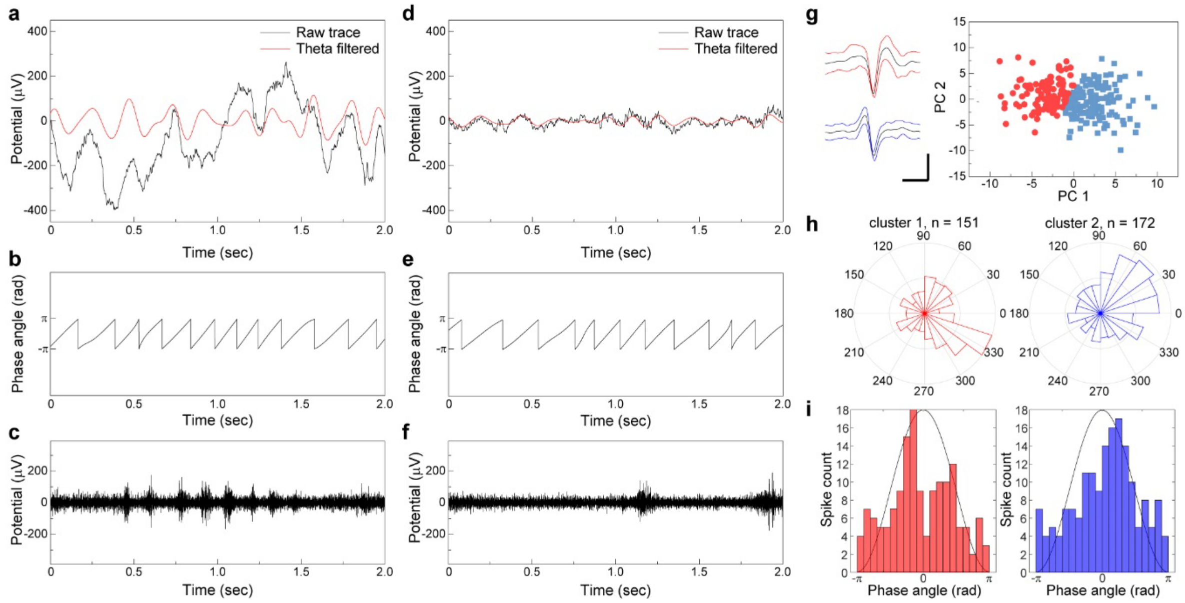

Supplementary Fig. 27. Hippocampal signals recorded from another mouse. **a-c,** Local field potential and superimposed theta waves (**a**), the corresponding theta angle (**b**), and single-unit trace (**c**) recorded from CA1 region during the active movement of mouse. **d-f,** Local field potential and superimposed theta waves (**d**), the corresponding theta angle (**e**), and single-unit trace (**f**) recorded from CA1 region during the standstill state of mouse. **g,** PCA-clustered single-unit spikes recorded in CA1 region. Scale bars, 100 µV (vertical) and 1 ms (horizontal). **h,** Circular distribution of theta angles for the firing events of each neuron recorded in CA1. **i,** Distribution of single-unit spikes to theta phase angle. Black lines represent a theta cycle**.**

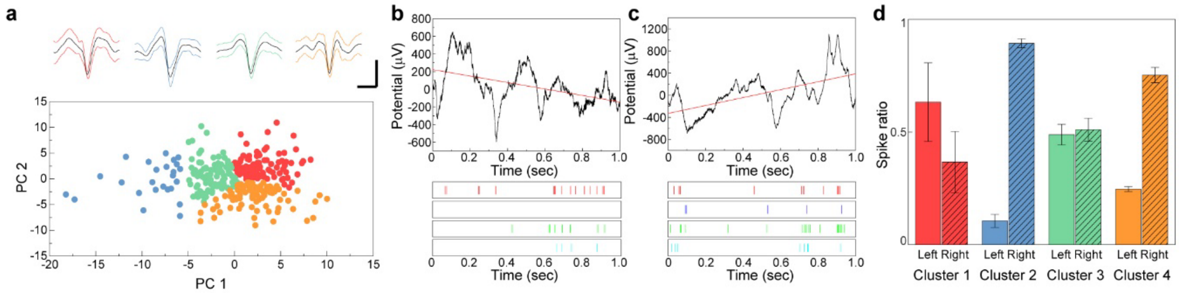

Supplementary Fig. 28. Motion-related signals from visual cortex of another mouse. **a,** PCA-clustered single-unit spikes recorded in L6 region. Scale bars, 100 μV (vertical) and 1 ms (horizontal). **b-c,** Representative local field potential and corresponding spike raster of PCA-clustered neurons during left-turn task of mouse (**b**) and right-turn task of mouse (**c**). Red lines are linear fit of LFP signals. **d,** Spike ratio of PCA-clustered neurons between left-turn and right-turn tasks for five times of trials. Error bars represent the standard deviation.

Supplementary Table

Reference	Materials	Number of channels	Subsidiary circuit	Recording area
Nature 551, 232 (2017)	TiN, Silicon (rigid)	960	PCBs (flat and rigid, wired)	Local (single-shank probe)
Nat. Neurosci. 25, 252 (2022)	TiN, Silicon (rigid)	384	PCBs (flat and rigid, wired)	Local (single-shank probe)
Nat. Commun. 12, 492 (2021)	Pt, Silicon (rigid)	63	PCBs (flat and rigid, wired)	Local (multi-shank probes)
Nat. Commun. 13, 5521 (2022)	Pt, Silicon (rigid)	16	PCBs (flat and rigid, wireless)	Local (single-shank probe)
Nat. Commun. 12, 3435 (2021)	Sn, hydrogel (flexible)	7	PCBs (flat and rigid, wired)	Local (fiber-based probe)
Nat. Mater. 18, 510 (2019)	Pt, Gold, SU-8 (flexible)	16	PCBs (flat and rigid, wired)	Local (syringe-injectable mesh electrodes)
Nat. Commun. 11, 6115 (2020)	BiSn, PC/PVDF (flexible)	4	PCBs (flat and rigid, wired)	Local (fiber-based probe)
Sci. Adv. 6, eaay2789 (2020)	PtIr, Parylene-C (flexible)	251	PCBs (flat and rigid, wired)	Widespread (random position)
Our work	EGaIn/Pt, Parylene-C (flexible)	12	Direct-printed on cranium (conformal, wireless)	Widespread (designated arbitrary position)

Supplementary Table 1. Comparison of our neural interface system to state-of-art neural probes.
