# Supplementary material for: In-vivo integration of soft neural probes through high-resolution printing of liquid electronics on the cranium
Source: Nat Commun. 2024 Feb 27;15:1772. doi: 10.1038/s41467-024-45768-0 (PMC10899244; doi:10.1038/s41467-024-45768-0)
Supplement: Supplementary file 3 — Description of additional supplementary files [file 41467_2024_45768_MOESM3_ESM.pdf]

DESCRIPTION OF ADDITIONAL SUPPLEMENTARY FILES DOCUMENT

Supplementary Movie 1. Conformal printing of liquid metal on the cranial surface of a mouse.

Supplementary Movie 2. Wireless reading of text information which is recorded on cranial circuits through a smartphone.

Supplementary Movie 3. The NFC-based wireless neural recording of a live mouse with cranial circuits through a smartphone, and the recovery of its healthy movement after the anesthetization wore off.

Supplementary Movie 4. Free moving of mouse in T-maze with wi-fi-based soft neural interface system.

Supplementary Movie 5. Monitoring of the heat generation by the cranial circuit during the T-maze test.
