# Supplementary material for: In-vivo integration of soft neural probes through high-resolution printing of liquid electronics on the cranium
Source: Nat Commun. 2024 Feb 27;15:1772. doi: 10.1038/s41467-024-45768-0 (PMC10899244; doi:10.1038/s41467-024-45768-0)
Supplement: Supplementary file 9 — Reporting Summary [file 41467_2024_45768_MOESM9_ESM.pdf]

nature portfolio

Corresponding author(s): Jang-Ung Park

Last updated by author(s): Jan 11, 2024

Reporting Summary

Nature Portfolio wishes to improve the reproducibility of the work that we publish. This form provides structure for consistency and transparency in reporting. For further information on Nature Portfolio policies, see our Editorial Policies and the Editorial Policy Checklist.

Statistics

For all statistical analyses, confirm that the following items are present in the figure legend, table legend, main text, or Methods section.

n/a	Confirmed	
☐	☒	The exact sample size (*n*) for each experimental group/condition, given as a discrete number and unit of measurement
☐	☒	A statement on whether measurements were taken from distinct samples or whether the same sample was measured repeatedly
☐	☒	The statistical test(s) used AND whether they are one- or two-sided<br>*Only common tests should be described solely by name; describe more complex techniques in the Methods section.*
☒	☐	A description of all covariates tested
☒	☐	A description of any assumptions or corrections, such as tests of normality and adjustment for multiple comparisons
☐	☒	A full description of the statistical parameters including central tendency (e.g. means) or other basic estimates (e.g. regression coefficient) AND variation (e.g. standard deviation) or associated estimates of uncertainty (e.g. confidence intervals)
☐	☒	For null hypothesis testing, the test statistic (e.g. *F*, *t*, *r*) with confidence intervals, effect sizes, degrees of freedom and *P* value noted<br>*Give P values as exact values whenever suitable.*
☒	☐	For Bayesian analysis, information on the choice of priors and Markov chain Monte Carlo settings
☒	☐	For hierarchical and complex designs, identification of the appropriate level for tests and full reporting of outcomes
☒	☐	Estimates of effect sizes (e.g. Cohen's *d*, Pearson's *r*), indicating how they were calculated

Our web collection on statistics for biologists contains articles on many of the points above.

Software and code

Policy information about availability of computer code

Data collection	Synapse (Tucker-Davis Technologies, Inc.)<br>VersaStudio
Data analysis	Synapse (Tucker-Davis Technologies, Inc.)<br>Origin 2019b<br>Imaris 9.0 Image Analysis<br>ImageJ 1.53t<br>Other analyses were done with custom Matlab code. The source code is available under the license of CC0 at Code Ocean (https://doi.org/10.24433/CO.1696883.v1).

For manuscripts utilizing custom algorithms or software that are central to the research but not yet described in published literature, software must be made available to editors and reviewers. We strongly encourage code deposition in a community repository (e.g. GitHub). See the Nature Portfolio guidelines for submitting code & software for further information.

Data

Policy information about availability of data

All manuscripts must include a data availability statement. This statement should provide the following information, where applicable:

- Accession codes, unique identifiers, or web links for publicly available datasets
- A description of any restrictions on data availability
- For clinical datasets or third party data, please ensure that the statement adheres to our policy

The main data supporting the results in this study are available within the paper and its Supplementary Information. The datasets generated during the study are too large to be publicly shared. Source data are available at Figshare (https://doi.org/10.6084/m9.figshare.24112461). Source data are provided with this paper for reproducing all Figures in the manuscript and Supplementary Information.
We used the Allen Mouse Brain Atlas to identify and illustrate the brain's stereotaxic coordination (Allen Mouse Brain Atlas. Available from https://mouse.brain-map.org).

Research involving human participants, their data, or biological material

Policy information about studies with human participants or human data. See also policy information about sex, gender (identity/presentation), and sexual orientation and race, ethnicity and racism.

Reporting on sex and gender	The study did not involve human research participants.
Reporting on race, ethnicity, or other socially relevant groupings	The study did not involve human research participants.
Population characteristics	The study did not involve human research participants.
Recruitment	The study did not involve human research participants.
Ethics oversight	The study did not involve human research participants.

Note that full information on the approval of the study protocol must also be provided in the manuscript.

Field-specific reporting

Please select the one below that is the best fit for your research. If you are not sure, read the appropriate sections before making your selection.

☒ Life sciences ☐ Behavioural & social sciences ☐ Ecological, evolutionary & environmental sciences

For a reference copy of the document with all sections, see nature.com/documents/nr-reporting-summary-flat.pdf

Life sciences study design

All studies must disclose on these points even when the disclosure is negative.

Sample size	Sample sizes were determined by the number of biological and technical replicates necessary to observe the effect was real. The minimum animal numbers of neural recording experiments (n=3) were determined based on previous publications [Nat. Mater. 18, 510–517 (2019), Adv. Sci. 10, 2300220 (2023)]. The minimum animal numbers of immunostaining experiments (n=6) were determined based on previous publications [Nat. Commun. 12, 3435 (2021), Nat. Commun. 10, 3777 (2019)].
Data exclusions	Animals with surgical complications and that displayed health concerns during the post-surgery monitoring period were excluded.
Replication	At least three biologically independent experiments were performed in each case of this study, and all experimental findings were reliably reproduced at least three times.
Randomization	All samples were allocated randomly into experimental groups.
Blinding	Blinding was not relevant, because a blinding process wouldn't influence the sampling result.

Reporting for specific materials, systems and methods

We require information from authors about some types of materials, experimental systems and methods used in many studies. Here, indicate whether each material, system or method listed is relevant to your study. If you are not sure if a list item applies to your research, read the appropriate section before selecting a response.

Materials & experimental systems

n/a	Involved in the study	
☐	☒	Antibodies
☐	☒	Eukaryotic cell lines
☒	☐	Palaeontology and archaeology
☐	☒	Animals and other organisms
☒	☐	Clinical data
☒	☐	Dual use research of concern
☒	☐	Plants

Methods

n/a	Involved in the study	
☒	☐	ChIP-seq
☒	☐	Flow cytometry
☒	☐	MRI-based neuroimaging

Antibodies

Antibodies used

Mouse anti-FOX3 (1:500, Cat# SIG-39860, Lot# B323799, Clone 1B7, BioLegend)
Rat anti-GFAP (1:300, Cat# 13-0300, Lot# XH352435, Clone 2.2B10, Invitrogen)
Rabbit anti-Iba1 (1:300, Cat# PA5-27436, Lot# XK3754735, Invitrogen)
Donkey anti-mouse Alexa Fluor 488 (1:300, Cat# A-21202, Lot# 2428531, Invitrogen)
Donkey anti-rat Alexa Fluor 594 (1:300, Cat# A-21209, Lot# 2400917, Invitrogen)
Goat anti-rabbit Alexa Fluor 647 (1:300, Cat# A-32733, Lot# XJ359307, Invitrogen)

Validation

Mouse anti-FOX3 (1:500, SIG-39860, Lot# XXXXX, BioLegend) has been validated by the manufacturer. "Each lot of this antibody is quality control tested by formalin-fixed paraffin-embedded immunohistochemical staining." (https://www.biolegend.com/fr-fr/products/purified-anti-fox3-neun-antibody-10823?GroupID=BLG15643) Relevant citation: J. Neurosci. 25, 2518 (2005).
Rat anti-GFAP (1:300, 13-0300, Invitrogen) has been validated by the relevant citation. "13-0300 was used in Immunohistochemistry to conclude that RHO-ROCK signaling is critical for normal adult NSC and TAP movement and interactions, which are compromised with age, concomitant with the loss of regenerative ability." [Stem Cell Report 17, 245 (2022)]
Rabbit anti-Iba1 (1:300, PA5-27436, Invitrogen) has been validated by the relevant citation. "PA5-27436 was used in Immunohistochemistry to introduce astrocytes as an important component of pain gating by activation of Aβ-fibers, which thus exert nonneuronal control of pain." [Sci. Adv. 7, eabi6287 (2021)]
Donkey anti-mouse Alexa Fluor 488 (1:300, A-21202, Lot# 2428531, Invitrogen) has been validated by the manufacturer. "Immunofluorescent analysis of HuC/D (green) and MAP2 (red) on rat primary cortical neurons cultured for 28 days in the B-27 Plus Neuronal Culture System." (https://www.thermofisher.com/antibody/product/Donkey-anti-Mouse-IgG-H-L-Highly-Cross-Adsorbed-Secondary-Antibody-Polyclonal/A-21202)
Donkey anti-rat Alexa Fluor 594 (1:300, A-21209, Lot# 2400917, Invitrogen) has been validated by the manufacturer. "Immunofluorescence analysis of Donkey anti-Rat IgG (H+L) Secondary Antibody, Alexa Fluor 594 conjugate was performed using A549 cells stained with alpha Tubulin (YL1/2) Rat Monoclonal Antibody ... Donkey anti-Rat IgG (H+L) Secondary Antibody, Alexa Fluor 594 conjugate (Product # A-21209) was used at a concentration of 1µg/mL in phosphate buffered saline containing 0.2 % BSA for 45 minutes at room temperature, for detection of alpha Tubulin in the cytoplasm." (https://www.thermofisher.com/antibody/product/Donkey-anti-Rat-IgG-H-L-Highly-Cross-Adsorbed-Secondary-Antibody-Polyclonal/A-21209)
Goat anti-rabbit Alexa Fluor 647 (1:300, A-32733, Lot# XJ359307, Invitrogen) has been validated by the manufacturer. "Multiplexed fluorescent western blot was performed Goat anti-Rabbit IgG (H+L) Highly Cross-Adsorbed Secondary Antibody, Alexa Fluor™ Plus 647 (Product # A32733)." (https://www.thermofisher.com/antibody/product/Goat-anti-Rabbit-IgG-H-L-Highly-Cross-Adsorbed-Secondary-Antibody-Polyclonal/A32733)

Eukaryotic cell lines

Policy information about cell lines and Sex and Gender in Research

Cell line source(s)

SH-SY5Y was purchased from Korean Cell Line Bank (KCLB 22266, Lot# 51972). Neuro2a was purchased from ATCC (CCL-131).

Authentication

Cell line was authenticated by company that we ordered from. Morphology check by microscope periodically. Cell morphology was similar to published pictures.

Mycoplasma contamination

The cell was used under mycoplasma-free condition.

Commonly misidentified lines
(See ICLAC register)

No commonly misidentified cell lines were used in this study.

Animals and other research organisms

Policy information about studies involving animals; ARRIVE guidelines recommended for reporting animal research, and Sex and Gender in Research

Laboratory animals

C3H mice (male, 6 weeks, 18~24 g, total 30 mice) and C57BL/6 mice (male, 6 weeks, 20~25 g, total 15 mice) were purchased from the Central Lab. Animal Inc. (Republic of Korea). The mice were raised in a specific pathogen free (SPF) environment with an ambient temperature of 23°C, a humidity of 50%, and a 12 h dark/light cycle.

Wild animals

The study did not involve wild animals.

Reporting on sex	Sex was not considered in the study, and only male mice was used for controlling the variables of the experiment.
Field-collected samples	The study did not involve samples collected from the field.
Ethics oversight	The animal experiments were performed under approval by the Committee on the Ethics of Animal Experiments of Yonsei University (IACUC-A-202011-1180-01).

Note that full information on the approval of the study protocol must also be provided in the manuscript.

Plants

Seed stocks	The study did not involve plant samples.
Novel plant genotypes	The study did not involve plant samples.
Authentication	The study did not involve plant samples.
